# PrP^Sc ^accumulation in neuronal plasma membranes links Notch-1 activation to dendritic degeneration in prion diseases

**DOI:** 10.1186/1750-1326-5-6

**Published:** 2010-01-21

**Authors:** Stephen J DeArmond, Krystyna Bajsarowicz

**Affiliations:** 1 Department of Pathology, University of California San Francisco, 1855 Folsom Street MCB 269, San Francisco, CA 94143-0803, USA; 2 Institute for Neurodegenerative Diseases, 1855 Folsom Street MCB 505, University of California San Francisco, San Francisco, CA 94143-0803, USA

## Abstract

Prion diseases are disorders of protein conformation in which PrP^C^, the normal cellular conformer, is converted to an abnormal, protease-resistant conformer rPrP^Sc^. Approximately 80% of rPrP^Sc ^accumulates in neuronal plasma membranes where it changes their physical properties and profoundly affects membrane functions. In this review we explain how rPrP^Sc ^is transported along axons to presynaptic boutons and how we envision the conversion of PrP^C ^to rPrP^Sc ^in the postsynaptic membrane. This information is a prerequisite to the second half of this review in which we present evidence that rPrP^Sc ^accumulation in synaptic regions links Notch-1 signaling with the dendritic degeneration. The hypothesis that the Notch-1 intracellular domain, NICD, is involved in prion disease was tested by treating prion-infected mice with the γ-secretase inhibitor (GSI) LY411575, with quinacrine (Qa), and with the combination of GSI + Qa. Surprisingly, treatment with GSI alone markedly decreased NICD but did not prevent dendritic degeneration. Qa alone produced near normal dendritic trees. The combined GSI + Qa treatment resulted in a richer dendritic tree than in controls. We speculate that treatment with GSI alone inhibited both stimulators and inhibitors of dendritic growth. With the combined GSI + Qa treatment, Qa modulated the effect of GSI perhaps by destabilizing membrane rafts. GSI + Qa decreased PrP^Sc ^in the neocortex and the hippocampus by 95%, but only by 50% in the thalamus where disease was begun by intrathalamic inoculation of prions. The results of this study indicate that GSI + Qa work synergistically to prevent dendrite degeneration and to block formation of PrP^Sc^.

## Introduction

Despite major advances in understanding prion biology and the conversion of PrP^C ^to PrP^Sc^, the mechanisms of early synaptic degeneration have remained largely unknown until now. In this review we show that accumulation of the abnormal prion protein (PrP^Sc^) in neuronal plasma membranes during prion diseases directly affects the processing of γ-secretase complex substrates and cause early dendritic degeneration.

The prion diseases in humans include sporadic Creutzfeldt-Jakob disease (sCJD), familial CJD (fCJD), and variant CJD (vCJD), the last acquired by ingestion of bovine spongiform encephalopathy (BSE)-contaminated food. Other human prion diseases include a thalamic variant of CJD called sporadic and familial fatal insomnia; kuru, acquired by ritualistic cannibalism in aborigines of New Guinea; and Gerstmann-Sträussler-Scheinker syndrome, a rare familial form of cerebral PrP amyloidosis. In animals, the main prion diseases include scrapie in sheep, which can be transmitted to rodents as laboratory scrapie; BSE in cattle; and chronic wasting disease in deer and elk.

Prion diseases are disorders of protein conformation in which PrP^C^, the normal cellular conformer, is converted to an abnormal, protease-resistant conformer named PrP^Sc^. PrP^Sc ^was first described in scrapie-infected Syrian hamsters in 1982 [1]. By convention, the abnormal prion protein is designated PrP^Sc ^in all prion diseases. sCJD is the most common prion disease, accounting for ~85% of the total human prion cases. In sCJD, spontaneous conversion of PrP^C ^to PrP^Sc ^begins the process of sustained conversion of PrP^C ^to PrP^Sc^. In fCJD, disease is caused by mutations of the prion protein gene (PRNP) [2]. Fewer than 1% of CJD cases are acquired by infection with exogenous prions such as prion-contaminated human growth hormone preparations, human dura mater grafts, corneal transplants, and BSE contaminated food products. In all cases of laboratory scrapie, disease is begun by inoculation with prions, usually injected unilaterally into the thalamus. As a general rule, the prions that are used in the laboratory are obtained from a 1:10 dilution of homogenized scrapie-infected mouse or hamster brain. In animals, injection of synthetic PrP^Sc ^manufactured in E. coli also produce disease verifying that PrP^Sc ^protein is the sole component of prions [3,4].

Plasma membrane dysfunction and degeneration occur very early in prion diseases, before the defining neuropathological change of vacuolar degeneration of gray matter. This vacuolation (spongiform degeneration) is an intermediate step in the morphological progression of prion disease and it precedes nerve cell death, which is a late morphological change. The importance of the plasma membrane in prion diseases is emphasized by the following data. More than 80% of the abnormal prion protein, PrP^Sc^, accumulates in the plasma membrane [5,6]. Many of the signs and symptoms of prion diseases are caused by dysfunction and degeneration of specialized portions of the membrane such as synapses [5,7,8]. The importance of plasma membrane degeneration in disorders such as CJD, kuru and scrapie was first recognized by the late Peter Lampert in 1969 [9,10]. Using electron microscopy, he found multiple foci of neuronal membrane degeneration and no defining viral particles. Therefore, he proposed that the plasma membrane is the main target of these diseases. Twelve years later, Stanley Prusiner found that the scrapie agent is composed of a single, abnormal, protease-resistant mammalian protein, the prion protein (PrP), and devoid of nucleic acid [1]. He named this unusual agent a "prion" and the mammalian protein that comprises the prion, PrP^Sc^. In this article, we review the evidence that PrP^Sc ^accumulates in high concentration in membrane rafts, activates Notch-1 signaling and, in doing so, promotes neuronal dendritic degeneration.

## Exponential increase of PrP^Sc^

Prion diseases are disorders of protein conformation. The normal cellular form of the protein, PrP^C^, is expressed constitutively at high levels in neurons and at low levels in glial cells [11]. PrP^C ^appears to be neuroprotective and protease sensitive. It's C-terminal half contains 3α-helices, A, B and C and it is attached to the outer leaf of the plasma membrane by a glycolipid anchor. The N-terminal half is largely unstructured. Like many glycolipid-anchored proteins, a high concentration of PrP^C ^is found in membrane rafts [12]. In CJD and scrapie, a protease resistant form of PrP accumulates, PrP^Sc^. It is believed that in PrP^Sc ^a large β-helix forms in the middle of the PrP^C ^molecule eliminating all of α-helix A and a portion of α-helix B [13]. PrP^Sc ^causes neurodegeneration. Multiple variations in a host animal's amino acid sequence of PrP^Sc^, variations in the β-structure imposed on PrP^Sc^, and differences in oligomerization of PrP^Sc ^determine the incubation time of the disease, the neuroanatomical distribution of vacuolar degeneration and the host species barrier that classically define prion strains [14].

How does the abnormal prion protein propagate? Fred Cohen suggested a model for the conversion of PrP^C ^to PrP^Sc ^in which he showed that when PrP^C ^is exposed to PrP^Sc ^it acquires an identical beta structure as PrP^Sc ^[15]. The process is not reversible. The conversion of the structure from α-helix to β-helix, or in some cases to a β-sheet is efficient, rapid and leads to an exponential increase in PrP^Sc^.

## PrP^Sc ^accumulates early in plasma membranes

The kinetics of PrP^Sc ^accumulation in subcellular fractions was measured during the course of scrapie in the thalamus and neocortex of Syrian hamsters inoculated unilaterally in the thalamus with the Sc237 strain of scrapie prions. The incubation time, defined as the time required to develop clinical signs of scrapie, is ~65 days in this model. Plasma membrane, endosomes and lysosomes were enriched in Percoll gradients and identified with specific markers [5]. The separation of subcellular fractions was accurate since plasma membrane, early and late endosomal, and lysosomal markers were highly enriched in the respective fractions. For example, Na-K ATPase was greatly enriched in plasma membrane fractions in control and scrapie infected Syrian hamsters. Endosomal fractions contained only ~20% of that found in membrane fractions, and none was found in lysosomes [5]. The volume and total protein of each fraction were monitored throughout the separation procedure in order to calculate the total Proteinase K (PK) resistant PrP^Sc ^in each fraction. A conformation-dependent immunoassay (CDI) was used to measure the amount of PrP27-30, the PK resistant peptide of PrP^Sc^, in the samples [16,17]. The CDI uses sodium-magnesium phosphotungstate to specifically precipitate PrP^Sc ^and separate it from PrP^C^. About 87 amino acids are removed from the N-terminal and a small portion of the C-terminal of PrP^Sc ^(32-35 kD) by PK digestion to yield the PrP27-30 peptide. The experiment described below was performed before we understood that ~50% of PrP^Sc ^in a Syrian hamster inoculated with Sc237 prions is PK sensitive (sPrP^Sc^) [18]. Evidence indicates that sPrP^Sc ^converts PrP^C ^to protease resistant PrP^Sc ^(rPrP^Sc^). Nevertheless, most of the evidence indicates that rPrP^Sc ^causes the neurological dysfunction and neurodegeneration in prion diseases, as we shall describe in this review.

**Figure 1:**
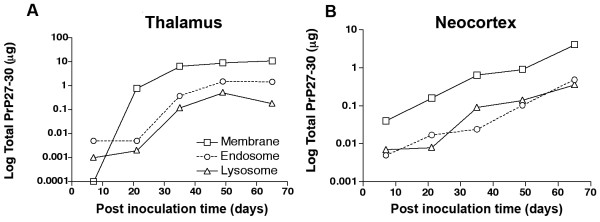
**Subcellular kinetics of log total PrP27-30 (μg) in the thalamus (**A**) and neocortex (**B**) throughout the course of Sc237 scrapie in Syrian hamster**. Hamsters were inoculated intrathalamically. PrP27-30 was measured by conformation-dependent immunoassay. Percoll gradient fractions: "plasma" membrane fraction; endosomal fraction; lysosomal fraction.

The great majority of rPrP^Sc ^was found in plasma membrane, endosomal and lysosomal fractions. Only a minor amount was found outside these three fractions (< 2%) (Fig. 1). Approximately 10-20 times more PrP^Sc ^accumulated in the plasma membrane in both thalamus (Fig. 1A) and neocortex (Fig. 1B), as compared to endosomes and lysosomes. A significant difference in the kinetics of PrP27-30 was seen in the thalamus and neocortex when the log of total PrP27-30 was plotted as a function of postinoculation time. In the thalamus, where disease was initiated by unilateral inoculation of PrP^Sc^, the conversion of PrP^C ^to rPrP^Sc ^(PrP27-30) began unilaterally and then spread bilaterally. PrP27-30 was detected in the endosomal and lysosomal fractions before it was detected in the plasma membrane fractions (Fig. 1A). The latter is consistent with the evidence that the conversion of PrP^C ^to PrP^Sc ^begins in the endocytic pathway in caveolae-like domains followed by deposition in lysosomes [19,20]. By 20 days postinoculation (dpi) 100 times more PrP27-30 accumulated in plasma membranes compared to endosomes and lysosomes. From 35-65 dpi, levels of PrP27-30 in the plasma membrane, endosomes and lysosomes reached a maximum with the level in membranes being the highest. From this data it appears that exogenous PrP^Sc ^in the inoculum undergoes reactive endocytosis to endosomes where it induces the conversion of PrP^C ^to PrP^Sc^. The sequence of events in the neocortex is different (Fig. 1B). First, rPrP^Sc ^was found in all subcellular fractions at the same time and the total amount of plasma membrane rPrP^Sc ^remained 10-20 times higher than levels in endosomes and lysosomes at every time point. This suggests that the conversion of PrP^C ^to PrP^Sc ^took place in both the plasma membrane and endosomes simultaneously (note: PrP^C ^is protease sensitive and, therefore, does not exist in lysosomes). Alternatively the conversion took place only in endosomes and transport to the plasma membrane occurred in a recycling pathway at a rate fast enough to hide it from our analysis. And second, levels of rPrP^Sc ^in the three subcellular compartments in the neocortex showed a simple exponential increase.

**Figure 2:**
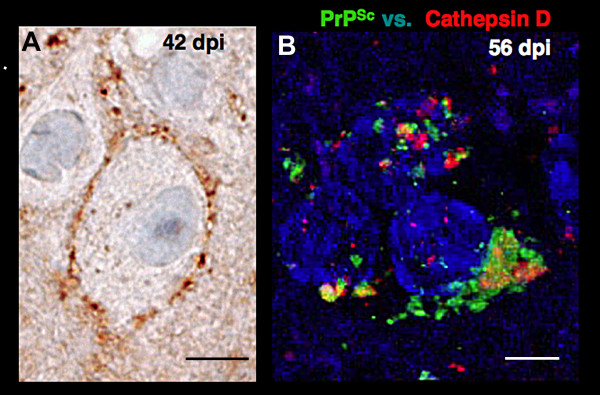
**Immunohistochemical localization of PrP^Sc ^in neurons of the neocortex in Syrian hamsters inoculated with the Sc237 prion strain**. **A**. A neuron in layer 4 of the neocortex at 42 days postinoculation (dpi) shows the plasma membrane filled with PrP^Sc^. Peroxidase immunohistochemistry with the PrP-specific 3F4 monoclonal antibody. **B**. Three neurons at 58 dpi show early endosomal, late endosomal, and lysosomal staining for PrP^Sc ^(green). Goat polyclonal IgG against Cat D (red) was obtained from Santa Cruz Biosciences. Nuclei are stained blue. See text for explanation. Bars in **A **represents 10 μm and in **B**, 5 μm.

In neurons, the accumulation of rPrP^Sc ^in the plasma membrane causes severe synaptic dysfunction early in the course of disease and before the nerve cell death [7,8,21]. Accumulation of PrP^Sc ^early in the plasma membrane of neocortical neurons is clearly demonstrated by peroxidase immunohistochemistry at 42 days in the hamster model (Fig. 2A). The membrane is filled with PrP^Sc^. Only a small amount of PrP^Sc ^can be seen in endosomes; however, the results with endosomes were not quantified because the method to enhance plasma membrane staining tended to preclude staining of endosomes and lysosomes. rPrP^Sc ^in endosomes and lysosomes is seen by double immunolabeling in confocal microscopy at 56 days in the Syrian hamster (Fig. 2B). We labeled PrP^Sc ^with green and Cat D with red fluorescent dyes. Pretreatment of the slide with autoclaving was adjusted to enhance endosomal and lysosomal staining, although it was too harsh for plasma membrane staining [22]. Three neurons at different stages of endocytosis were stained. In the uppermost neuron, PrP^Sc ^is seen in early endosomes. Cat D, which is characteristic of late endosomes and lysosomes, is largely separated from early endosome-containing PrP^Sc^, although a few show a degree of overlap with Cat D (yellow), especially in the bottom left neuron. In the bottom right neuron, the compartments containing PrP^Sc ^and Cat D have merged and formed one large dystrophic-like structure at the base of the neuron that resembles an autophagic vesicle. Electron microscopy has confirmed that these aggregates are indeed autophagosomes [23-25]. Large autophagic vacuoles precede or co-exist with nerve cell death at virtually all stages of scrapie in mice, but they are most prominent in the late stage of prion disease due to the exponential increase of neurons forming rPrP^Sc ^[26]. It is not clear whether autophagy plays a disease promoting role or a disease defensive role since activation of autophagic vacuoles promotes degradation of PrP^Sc ^and a prolongation of incubation time [27]. Nevertheless, this change in neurons indicates a pre-death stage. When fixed with glutaraldehyde and paraformaldehyde and viewed in 1 μm thick plastic sections by light microscopy, neurons with autophagic vesicles appear dark (dark cell degeneration) [28]. In the hamster neocortex, cells with dark cell degeneration accounted for ~60% of the neurons at 70 dpi with prions; whereas, in age-matched controls they accounted for only ~10% [26]. At 42 dpi, only ~10% of neurons were dark neurons in comparison to less than 1% of neurons in age-matched controls. It is likely that a small number of neurons die at all stages of disease. The few neurons that die early are probably the neocortical neurons first infected by transynaptic spread of the conversion of PrP^C ^to rPrP^Sc ^whose lysosomes become filled with rPrP^Sc^. Some of them may be interneurons with short connections. The largest number of neocortical neurons reach a pre-death stage near the end of the disease when rPrP^Sc ^has spread exponentially to many neurons in all layers of the neocortex. A similar temporal sequence has been described in mice where synapse degeneration precedes nerve cell death by 1-2 months [7].

We focused on the early synaptic dysfunction and degeneration because these changes produce clinical symptoms and because it may be possible to intervene with drug therapy at this stage of the disease. Late-occurring neuronal death may be treatable with compounds that influence autophagy [27,29]. It should be noted that most CJD patients die at an intermediate stage of disease with vacuolization and little or no detectable nerve cell loss [30].

## rPrP^Sc ^is transported from one brain region to another by axonal transport

**Figure 3:**
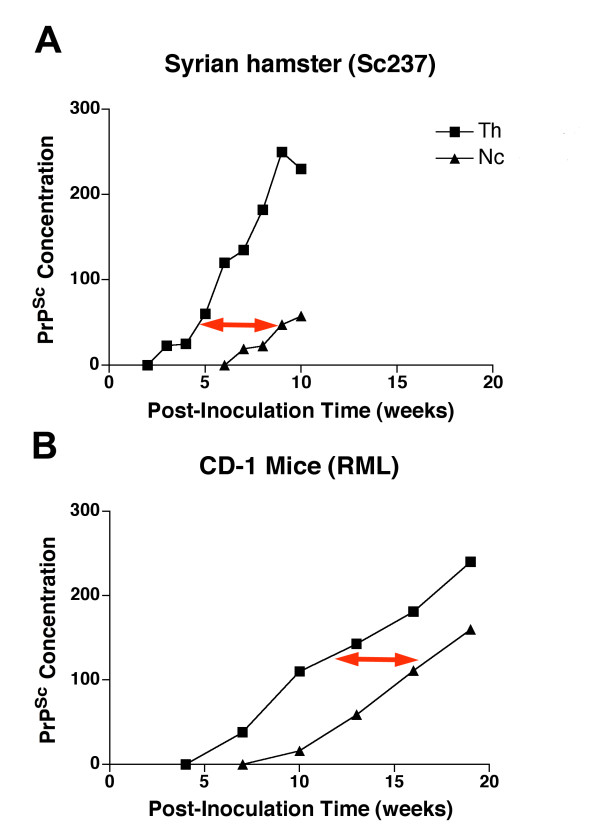
**PrP^Sc ^is transported along axons from one brain region to another**. Data from the thalamus (squares) and the neocortex (triangles) are shown. **A**. Syrian hamsters inoculated in the thalamus with the Sc237 prion strain become clinically ill at ~10 weeks. Measurements of rPrP^Sc ^were generated by Western blot analysis from dissected brain regions. **B**. CD1 mice inoculated in the thalamus with RML prions become clinically ill at ~20 weeks. rPrP^Sc ^was estimated by morphometry of brain regions on histoblots.

The first evidence that rPrP^Sc ^is transported from one brain region to another came from quantitative studies of dissected brain regions. The rate of transport of rPrP^Sc ^among brain regions was examined in two models of prion disease with two different incubation times. Syrian hamsters inoculated with Sc237 prion strain have an incubation time of ~10 weeks (Fig. 3A) [31] and CD1 mice inoculated with the Rocky Mountain Laboratorie's (RML) prion strain have an incubation time ~20 weeks (Fig. 3B) [32]. The earliest and highest accumulation of rPrP^Sc ^occurred in the thalamus in both models and it took approximately 4 weeks for transmission of rPrP^Sc ^from the thalamus to the neocortex in both models. The data suggests that rPrP^Sc ^spreads from one brain region to another by very slow axonal transport [33]. It also indicates that the rate of spread is independent of incubation time.

**Figure 4:**
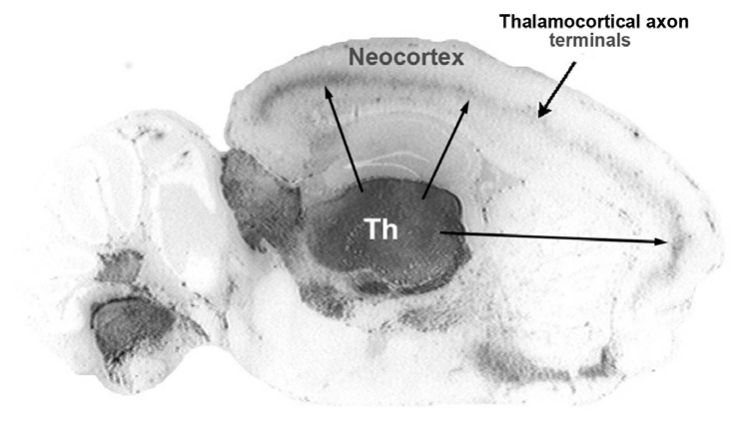
**Histoblot of Syrian hamster brain sagittal section at 49 dpi after inoculation with the Sc237 prion strain**. rPrP^Sc ^was stained with PrP-specific 3F4 antibodies. See text for explanation. Adapted from Bouzamondo-Bernstein et al. [5].

Axonal transport of rPrP^Sc ^is clearly demonstrated by histoblot analysis (Fig. 4). A 10 μm thick fresh frozen section of Sc237 scrapie-infected Syrian hamster brain was "blotted" onto nitrocellulose paper and treated with PK to eliminate PrP^C^. The remaining rPrP^Sc ^was detected with the PrP-specific antibodies following denaturation with guanidinium. At 49 dpi, a sagittal section showed the thalamus filled with rPrP^Sc ^(Fig. 4). At the same time, the neocortex contained linear deposits of rPrP^Sc ^in Layer 4, where the thalamocortical axons terminate. These deposits became visible when a sufficiently large number of presynaptic boutons and postsynaptic neurons acquired rPrP^Sc^.

This evidence suggests a very slow axonal transport system requiring ~4 weeks to travel 3-5 mm and precludes any of the active transport systems, which take only hours to days. We believe PrP^Sc ^diffuses passively along the axon membranes, driven by a concentration gradient that is higher near the cell body in the thalamus and is progressively lower towards neocortex [33]. Alternatively, as rPrP^Sc ^diffuses into axons it may convert adjacent axonal PrP^C ^molecules into sPrP^Sc ^or rPrP^Sc ^resulting in the continuous conversion process that eventually reaches presynaptic boutons.

## Axonal terminals and transynaptic conversion of PrP^C ^to PrP^Sc^

**Figure 5:**
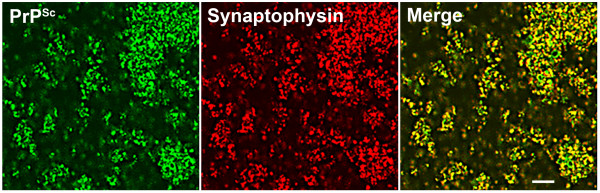
**Colocalization of total PrP^Sc ^(rPrP^Sc ^+ sPrP^Sc^) and synaptophysin in synaptosomes**. Confocal micrographs of a "crude" synaptosomal preparation obtained from the Sc237 prion strain infected hamsters at 70 dpi show total PrP^Sc ^(green) and synaptophysin (red) (mouse monoclonal anti-synaptophysin, clone SY38, DakoCytomation). Merged images show colocalization of total PrP^Sc ^and synaptophysin in yellow. Bar represents 20 μm. Adapted from Bouzamondo-Bernstein et al. [5].

Electron microscopy has shown that PrP^C ^is located ubiquitously throughout the plasma membrane, which includes synaptic boutons and postsynaptic membranes; PrP^C ^was not found on synaptic vesicles [34]. Since the ultrastructural location of rPrP^Sc ^is less well known, we must infer its location from synaptosomal immunohistochemistry (Fig. 5), from immunohistochemistry of tissue sections (Figs. 2A, 4) and from the kinetics of PrP^Sc ^(Fig. 3). It seems that rPrP^Sc ^is present in high concentrations on the surface of the presynaptic bouton, as the result of axonal transport [33].

To examine PrP^Sc ^accumulation in the presynaptic boutons we prepared synaptosomes from the neocortex of Syrian hamsters at 70 dpi. Synaptosomes, which contain presynaptic boutons and postsynaptic membranes, were stained for rPrP^Sc ^(green) and synaptophysin (red) [5] (Fig. 5). Synaptophysin is one of the specific markers for synaptic vesicles in presynaptic boutons. rPrP^Sc ^appears to be on the surface of the synaptosomes as well as in the interior (Fig. 5). The majority of the objects in the figure were also immunostained for synaptophysin as seen in the merged image (Fig. 5). The similarity of the immunohistochemistry for PrP^Sc ^and synaptophysin suggest that some PrP^Sc ^is located within the axon terminals. Not all of the PrP^Sc ^positive structures were synaptophysin positive. They probably represent fragments of plasma membrane contaminants.

The conversion of PrP^C ^to PrP^Sc ^begins in the postsynaptic membrane and continues to be formed in the postsynaptic neurons by one of two possible mechanisms: degeneration of the presynaptic bouton [5,21] or release of PrP^Sc ^during synaptic transmission. Once it traverses the synaptic cleft, it can bind to PrP^C ^on the postsynaptic membrane and convert it to rPrP^Sc^, possibly in endosomes by endocytosis of PrP^C^-PrP^Sc ^complex.

## Dendritic degeneration occurs early in prion disease

**Figure 6:**
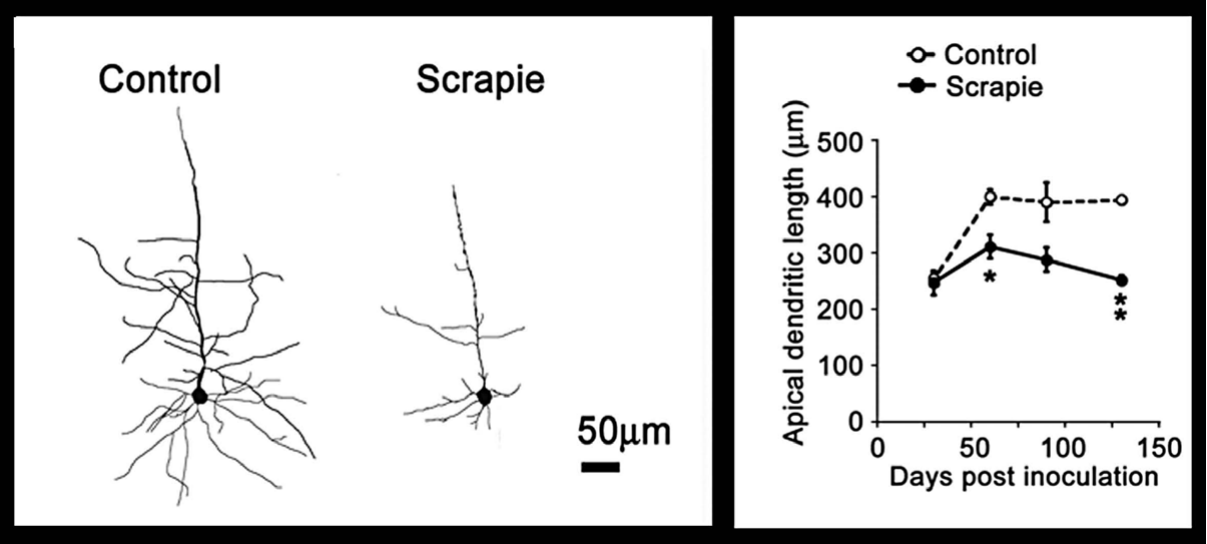
**Progressive degeneration of dendrites during prion disease occurs early in the incubation time **[35]. CD1 mice were inoculated in the thalamus with RML scrapie prions. **A**. Representative Golgi silver impregnation of dendrites in the neocortex show severe atrophy in scrapie-infected mice compared to controls. **B**. Apical dendritic length versus incubation time in age matched controls (open circles) and in scrapie (closed circles) is shown during the course of scrapie. Adapted from Ishikura et al. [35].

CD1 mice inoculated unilaterally in the thalamus with RML prions show early regressive changes in dendrites 70 to 80 days before the mice became clinically ill with scrapie at ~130 dpi [35]. At this time, dendrites of neocortical pyramidal neurons showed remarkable regressive changes in scrapie-infected mice compared to age-matched controls (Fig. 6A). We quantified the length of the pyramidal neuron dendrites in the neocortex throughout the course of scrapie and compared them with age-matched normal controls (Fig. 6B). At 30 dpi, the control and infected mice had similar dendrite lengths. Both control and infected animals showed dendrite growth as they matured, but in the infected animals the dendritic growth was much slower. From 50-130 dpi there was significant decrease in dendritic length in mice infected with prions, compared to age matched controls. The dendritic degeneration increased to 50% by 130 dpi. A similar finding was made for the number of dendritic branches (data not shown). It should be noted that the regressive dendritic changes began early in the neocortex, between 30 and 50 dpi, and before vacuolization, which occurred at 80 to 90 dpi. The data indicate that dendritic regressive changes are among the earliest morphological feature in prion disease. However, it is reported that C57BL/6 mice inoculated bilaterally in the hippocampus with the ME7 prion strain showed only decreased expression in proteins of the presynaptic compartment and degeneration of hippocampal presynaptic boutons and no dendritic degeneration [21]. The authors report intact dendritic cytoarchitecture based on the β-tubulin immunostaining; however, the lengths of dendrites and branching were not reported and their findings are difficult to assess from their photomicrographs.

## Notch-1 signaling in prion diseases

During embryonic development of the central nervous system, regressive changes in dendrites are regulated by a Notch-1 signaling pathway [36,37]. Activation of Notch-1 stimulates the HES and HERP families of inhibitor effecter proteins [38], which in turn inhibit pro-neuronal genes that maintain the length and branching of dendrites. During development, ligands such as Delta-like and Jagged expressed on adjacent cells lead to the truncation of Notch-1 [39]. The truncated Notch-1 is a specific ligand for Nicastrin, which is 1 of the 4 proteins that comprise γ-secretase complex. Nicastrin carries the truncated Notch-1 into the γ-secretase complex where it cleaves off the active molecule called the Notch-1 intracellular domain, NICD [40,41]. NICD is translocated to neuronal nuclei where it activates the HES and HERP genes that inhibit the expression of pro-neuronal genes that maintain dendrites. We speculated that if this pathway were active in prion disease, we might find increased amounts of NICD and perhaps changes in the γ-secretase complex itself.

**Figure 7:**
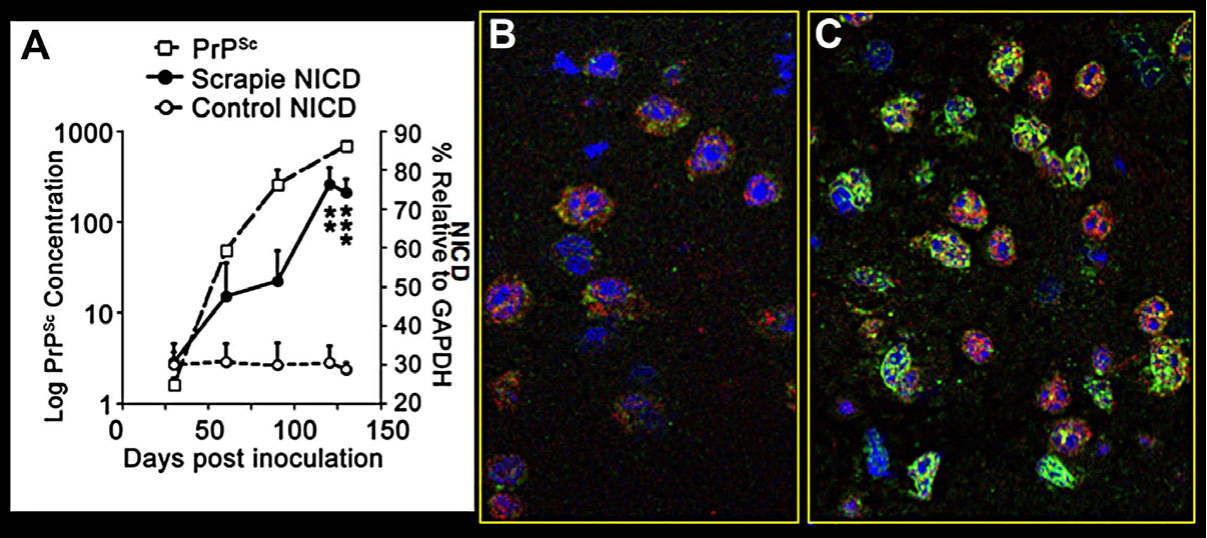
**rPrP^Sc ^accumulation in neocortical synaptosomes coincide with increased amounts of NICD and translocation of NICD to neuronal nuclei**. **A**. Kinetics of the log of neocortical rPrP^Sc ^accumulation in synaptosomes (open squares) relative to NICD concentration (filled circles). NICD levels in age matched PBS-inoculated mice are shown as controls (open circles). **B**. In control mice, confocal microscopy shows small amounts of NICD mostly in the cytoplasm in uninfected neocortical neurons. **C**. Confocal microscopy shows accumulation of NICD in the nuclei of cortical neurons in RML infected mice at 140 dpi. NICD (green) is detected by Notch-1 Val-1744 antibody from Cell Signaling Technology and the neuron specific antibody anti-NeuN (red) is from Chemicon. In **A**, data points are calculated from three independent experiments. ** P < 0.005; *** P < 0.0001 by Student's *t *test. Adapted from Ishikura et al. [35].

The first evidence that the Notch-1 pathway might be important in prion disease was the finding that the formation of NICD is associated with rPrP^Sc ^accumulation in neocortical synaptosomes (Fig. 7A) [35]. NICD increased rapidly between 30 and 50 dpi, which corresponded to the period when dendrites showed the first signs of regression (Fig. 6B). During that period the formation of NICD correlated well with rPrP^Sc ^accumulation in membranes of neocortical synaptosomes (Fig. 7A). The correlation suggested that rPrP^Sc ^was responsible for the truncation of Notch-1 rather than a ligand such as Delta-like or Jagged. In scrapie infected mice, NICD was translocated to the neuronal nucleus (Fig. 7C), whereas in control mice, low levels of NICD were located mostly in neuronal cytoplasm (Fig. 7B).

**Figure 8:**
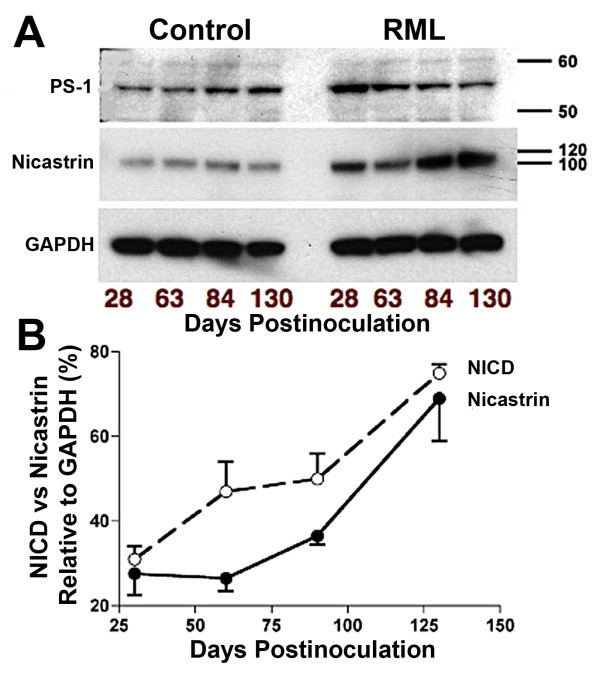
**Triphasic NICD response to rPrP^Sc ^accumulation corresponds with delayed increased expression of Nicastrin**. **A**. Western blots during the course of RML scrapie show decreased amount of PS-1 (Presenilin 1) and increased amount of Nicastrin relative to GAPDH standard. **B**. NICD and Nicastrin are plotted as a function of incubation time. The initial steep rise in NICD corresponds to rPrP^Sc ^accumulation, followed by relatively flat NICD level. The late occurring rise in NICD corresponds to increased expression of Nicastrin. n = 3 mice at each time point.

The increasing NICD curve was triphasic (Fig. 8B). After an initial rapid increase of NICD at 30-50 days, the NICD level plateaued between 50-90 days and then resumed a rapid increase at 90-130 days. Nicastrin, the molecule that transports truncated Notch-1 to the γ-secretase complex, showed a delayed increase, which corresponds to the late increase of NICD level from 90-130 dpi (Fig. 8A, B). This suggests that between 60 and 100 days truncated Notch-1 was being formed at a faster rate than Nicastrin could transport it to γ-secretase and that all of the available Nicastrin molecules had become saturated with truncated Notch-1. From 90-130 days, when Nicastrin levels increased 2 fold, the rate of formation of NICD increased accordingly. We do not know what caused the membrane concentration of Nicastrin to rise. Presenilin-1, the γ-secretase component that cleaves NICD from truncated Notch-1, showed a decrease or no change at all (Fig. 8A).

## Cholesterol rich membrane rafts: Hypothesis

In normal control brains PrP^C^, Notch-1 and γ-secretase are concentrated in membrane rafts [42]. Similarly, in prion infected brains PrP^Sc ^(sPrP^Sc ^and/or rPrP^Sc^), Notch-1 and γ-secretase are concentrated in membrane rafts. How then does PrP^Sc ^lead to formation of NICD? We can only guess at this time. The data suggest that PrP^Sc ^causes truncation of Notch-1 in a dose dependent manner, probably without participation of Delta-like or Jagged ligands. Nicastrin transports the truncated Notch-1 to the γ-secretase complex. From these data we propose a null hypothesis that inhibition of NICD formation during prions disease will prevent dendritic degeneration. As we shall see, this hypothesis fails to account for the complexity of γ-secretase functions.

## Treatment strategy

We obtained γ-secretase inhibitor (GSI) LY411575 from Drs. Todd Golde and Pritam Das at the Mayo Clinic, Jacksonville, Florida. This is a particularly potent Notch-1 inhibitor. Like many GSI's designed to treat Alzheimer's disease, it produces severe gastrointestinal tract and immune system toxicity; therefore, it is not used clinically. However, we believed it could be used for preclinical proof of concept studies. We also planned to combine quinacrine (Qa) with GSI because Qa was found to clear all PrP^Sc ^from scrapie-infected ScN2a cells within 5 days [43]. It was our belief that any reduction of PrP^Sc ^in brain caused by Qa would be additive to the GSI effect in terms of reducing NICD formation and dendrite degeneration.

Wild type CD1 mice were inoculated in the thalamus with RML prions. Oral doses of GSI alone (5 mg/kg/day), Qa alone (40 mg/kg/day) and combined GSI + Qa (same doses) were prepared in a nutritional chocolate drink containing 0.007% DMSO. The treatment was started at 50 dpi when disease was well established in the thalamus and had just begun to spread to the neocortex. Because of GSI toxicity, continuous treatment had to be terminated after 43 to 55 days.

## **Effect of treatment on dendrites**

**Figure 9:**
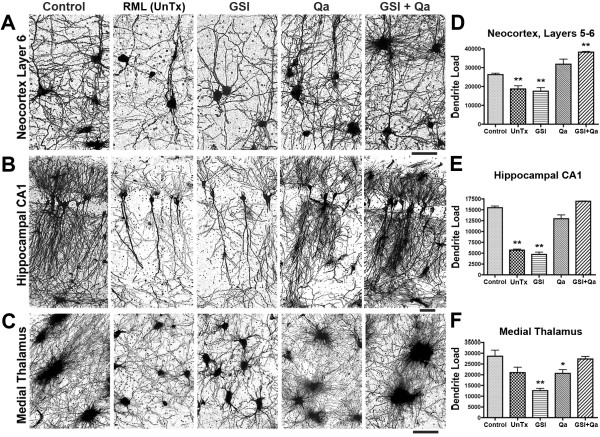
**Combined GSI + Qa treatment prevented dendritic degeneration more effectively than GSI or Qa alone**. **A-C**. Golgi silver staining in dendrites in layer 6 of the cortex (**A**), hippocampal CA1 region (**B**), and the medial nuclei of the thalamus (**C**). **D-F**. Bar graphs of the dendrites load in the three regions respectively as determined by morphometry. RML infected untreated mice (UnTx) shows severe dendritic atrophy. Prevention of dendritic loss was associated with GSI + Qa treatment. Measurements of dendrite loads in each of the three brain regions are shown as a function of the treatment. Student *t *test probabilities (n = 3 mice each): * P ≤ 0.05; **P ≤ 0.01. Scale bars represent 30 μm and apply to each image in the same row. Adapted from Spilman et al. [8].

Pharmacological reduction of PrP^Sc ^is clinically meaningful only if it prevents neurodegeneration. To quantify the dendrite loads, dendrites and nerve cell bodies were stained by the Golgi silver impregnation method. Measurements of dendrite loads were obtained from layer 6 of the neocortex, the CA1 region of the hippocampus and the medial thalamus (Fig. 9A-C). We measured the total number of dendrites and nerve cell bodies using BioQuant^® ^software (Fig. 9D-F). After 43 days, three age-matched control mice showed richly branched dendritic trees in all 3 regions. In untreated RML prion-infected mice, dendrite shortening and a decrease in the number of dendrite branches were severe in all 3 regions. Surprisingly, 43 days of treatment with GSI alone had no effect on the dendritic trees; they remained as atrophied as in untreated RML-infected animals. In contrast, Qa alone produced near normal dendritic trees. And, combined GSI + Qa treatment resulted in significantly more dendrites and a richer dendritic tree than controls. These results were unexpected to us. We thought GSI would have a very positive effect on dendrite lengths and number of branches. We believed Qa would produce no effect.

**Figure 10:**
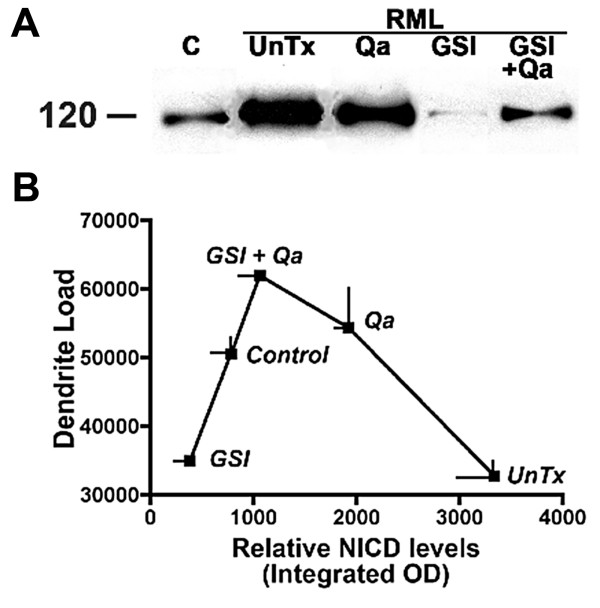
**The three treatments had significantly different effects on nuclear NICD levels and dendritic degeneration in the cortex**. **A**. Western blot analysis shows nuclear NICD levels from prion infected CD1 mice after treatment with Qa alone, GSI alone and Qa + GSI. For controls, nuclear NICD levels in an uninfected mouse (Control, C) and in an untreated RML prion infected mouse (UnTx) are shown. **B**. Relative NICD levels are plotted as a function of dendritic load (see Fig. 9D). See text for discussion. Error bars for both dendrite load and NICD (n = 3 mice each) are shown [8].

There are 20-30 substrates cleaved by the γ-secretase complex. Notch-1 is the best-known inhibitor of dendritic growth [44,45]. Two γ-secretase substrates that stimulate dendritic growth are ErbB-4 [46,47] and EphA4 [42]. Whether or not more stimulators and inhibitors will be found has yet to be determined. In the context of these considerations and to better understand our results we plotted dendrite load versus the concentration of NICD in neocortex (Fig. 10). For detection of NICD, nuclei were enriched using the Nuclear EZ prep according to kit instructions. Anti-NICD Val 1744 (Cell Signaling Technology) was used as a primary antibody for Western blots.

Control mice normally express a small amount of NICD (Fig. 10A). In the dendrite load versus NICD level plot, control NICD levels are associated with abundant dendrites indicating a balance between stimulators and inhibitors (Fig. 10B). In the untreated RML infected mice, a 3 to 4 fold increase of NICD was associated with a marked reduction in the dendrite load as seen in the graph. This suggests that prion infection shifted the balance toward inhibition. GSI alone was associated with a very low NICD level, as expected, but dendritic trees showed no recovery (Fig. 9), which argues that GSI inhibited both γ-secretase stimulators and inhibitors of dendritic growth. Combined GSI + Qa treatment resulted in an almost normal NICD level and a greater than control dendritic arborization (Fig. 10). This suggests that Qa was able to modulate the effect of GSI. Qa "destabilizes" membrane rafts by redistributing cholesterol from the plasma membrane to endosomes and lysosomes [48]. We speculate that perhaps Qa also separates PrP^Sc ^from Notch-1 or separates components of the γ-secretase complex from each other. Qa alone doubled NICD levels compared to controls and was associated with a near normal dendritic load (Fig. 10). To accomplish this, Qa must have also proportionately increased the stimulators of dendritic growth. The data demonstrate that GSI + Qa work synergistically to regulate the maintenance of dendrite size and shape in prion disease. Furthermore, these data argue that the γ-secretase complex plays a major role in the early dendritic changes.

## GSI + Qa treatment decreases rPrP^Sc^

**Figure 11:**
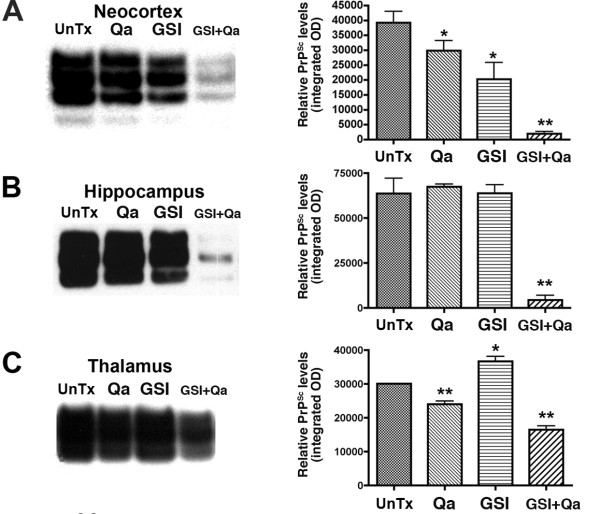
**Combined GSI + Qa therapy reduced rPrP^Sc ^in prion infected CD1 mice by 95% in the neocortex and hippocampus and by 50% in the thalamus**. Western immunoblots of rPrP^Sc ^in the neocortex (**A**), hippocampus (**B**), and thalamus (**C**) are shown. Adjacent bar graphs show densitometry estimates of the relative rPrP^Sc ^levels in the respective Western blots (integrated optical density units per milligram of total protein). Means and standards deviation are shown. Student's *t *test probability (n = 3 mice each): * P ≤ 0.05; ** P ≤ 0.01. Adapted from Spilman et al. [8].

**Figure 12:**
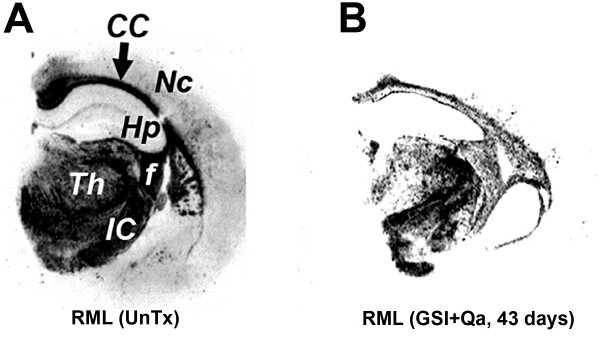
**rPrP^Sc ^shown in histoblots of coronal sections through the dorsal hippocampus where it overlies the thalamus**. Samples were taken from RML-infected mice at 93 dpi untreated (**A**) and treated with GSI + Qa for 43 days (**B**). CC, corpus callosum; f, fimbria of fornix; Hp, hippocampus; IC, internal capsule; Nc, neocortex; Th, thalamus. Adapted from Spilman et al. [8].

GSI + Qa resulted in a 95% decrease of rPrP^Sc ^in neocortex and hippocampus (Fig. 11A, B). In the thalamus, where prion replication was initiated, GSI + Qa reduced rPrP^Sc ^levels by only ~50% (Fig. 11C). This suggested that ~50% of rPrP^Sc ^in the thalamus could not be cleared by GSI + Qa. Histoblot showed that most of the residual rPrP^Sc ^was located in white matter tracts in and around the thalamus (Fig. 12). In untreated scrapie at 43 days (93 dpi), PrP^Sc ^fills the entire thalamus including the grey and white matter, and lower amounts of rPrP^Sc ^are also found in the hippocampus and neocortex (Fig. 12A). In addition, rPrP^Sc ^is located in long tracts connecting the thalamus with other regions. With GSI + Qa treatment for 43 days, most of rPrP^Sc ^has been cleared from the grey matter of the thalamus as well as from the hippocampus and neocortex (Fig. 12B). Most remaining rPrP^Sc ^is located in white matter tracts passing through the thalamus and those connecting the thalamus with the other regions. In the thalamus where the disease was started, it appears that some rPrP^Sc ^entered the white matter before treatment was begun.

## Residual rPrP^Sc ^in white matter can restart the disease

**Figure 13:**
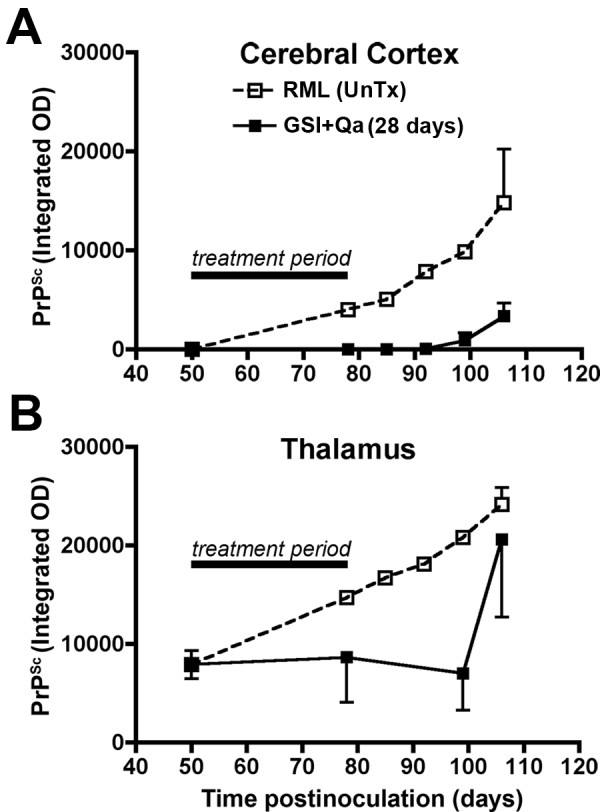
**After 28 days of treatment with GSI + Qa, rPrP^Sc ^levels remain low for an additional 21 days after treatment is ceased**. Estimates of rPrP^Sc ^concentration (n = 3 mice) in the neocortex (**A**) and thalamus (**B**) are given. Relative PrP^Sc ^levels from untreated infected mice at 78, 85, 92 and 99 dpi were estimated by using data from an earlier study [32]. Adapted from Spilman et al. [8].

In a second GSI experiment, mice were treated with GSI + Qa for 28 days, which was sub lethal (Fig. 13). We found that rPrP^Sc ^was cleared from the grey matter of the thalamus within a day or two of the start of treatment (Fig. 13B), but low levels of rPrP^Sc ^persisted in the white matter tracts during treatment and for 21 days thereafter. A sharp rebound of the conversion of PrP^C ^to rPrP^Sc ^in the thalamus (Fig. 13B) and to a lesser extent in the neocortex (Fig. 13A) began 21 days after the end of treatment, which suggested that the surviving rPrP^Sc ^was able to restart the disease. It also appears that GSI + Qa altered or greatly decreased axonal transport of rPrP^Sc ^to other brain regions based on the response in the neocortex, which only showed a slight increase in rPrP^Sc ^at 21 days after the end of treatment. The implication of these findings is that a shorter period of oral treatment with the GSI (LY411575) + Qa is tolerable and can hold the disease in check. New GSIs with less toxicity may become available, which will permit longer treatment. In the meantime, we intend to explore whether a short oral course of GSI + Qa in combination with gene therapy could stop disease progression. Adding concomitant gene therapy to the thalamus with a short interfering RNA (siRNA) against PrP^C ^theoretically will give long-term control of the disease by removing the substrate for rPrP^Sc ^formation.

## Conclusions

In this review we focused on the exponential accumulation of rPrP^Sc ^in the plasma membrane of neurons and how it causes regressive changes in dendrites. We did not review the additional evidence from our laboratory that rPrP^Sc ^was associated with decreased evoked release of neurotransmitters from neocortical synaptosomal preparations during scrapie in Syrian hamsters and that it was associated with presynaptic bouton degeneration [5]. In other experiments, accumulation of rPrP^Sc ^in membranes in mouse scrapie decreased binding of specific ligands to receptors on the postsynaptic membrane [49,50]. Accumulation of rPrP^Sc ^in the plasma membrane of a scrapie-infected N2a cell line (ScN2a) was found to decrease membrane fluidity by 7-fold compared to uninfected N2a cells [51,52]}. A similar change in neuronal membrane properties was reported for synaptosomal membranes [53]. These data argue that the early accumulation of rPrP^Sc ^in neuronal plasma membranes should not be ignored. Finally, the importance of lipid raft domains must be emphasized because γ-secretase [42], Notch-1, and the glycolipid-anchored proteins (PrP^C^, and PrP^Sc^), all are concentrated within rafts. This most likely explains why quinacrine and the γ-secretase inhibitor work synergistically to decrease rPrP^Sc ^and to prevent dendritic degeneration. The data suggest that dendritic degeneration is mediated by the γ-secretase complex. Furthermore, the response of PrP^Sc ^to GSI + Qa raises the possibility that the conversion of PrP^C ^to PrP^Sc ^is also mediated, at least in part, by the γ-secretase system.

## Competing interests

The authors declare that they have no competing interests.

## Authors' contributions

The review covers 24 years of research by SD, in the DeArmond Neuropathology Research Laboratory. He has directed all the research described in this paper. KB, who directs the molecular and cell biology research in the DeArmond Lab, is very knowledgeable about the biology of prion diseases. Both authors contributed equally to writing the text.
